# Identification of microRNAs involved in pathways which characterize the expression subtypes of NSCLC

**DOI:** 10.1002/1878-0261.12571

**Published:** 2019-09-22

**Authors:** Ann Rita Halvorsen, Miriam Ragle Aure, Åsa Kristina Õjlert, Odd Terje Brustugun, Steinar Solberg, Daniel Nebdal, Åslaug Helland

**Affiliations:** ^1^ Department of Cancer Genetics Institute for Cancer Research OUS Radiumhospitalet Oslo Norway; ^2^ Institute for Clinical Medicine University of Oslo Norway; ^3^ Department of Cardiothoracic Surgery Oslo University Hospital‐Rikshospitalet Norway

**Keywords:** adenocarcinoma, expression subtypes, microRNA, non‐small‐cell lung cancer, pathway, predicted target, squamous cell carcinoma

## Abstract

Dysregulation of microRNAs is a common mechanism in the development of lung cancer, but the relationship between microRNAs and expression subtypes in non‐small‐cell lung cancer (NSCLC) is poorly explored. Here, we analyzed microRNA expression from 241 NSCLC samples and correlated this with the expression subtypes of adenocarcinomas (AD) and squamous cell carcinomas (SCC) to identify microRNAs specific for each subtype. Gene set variation analysis and the hallmark gene set were utilized to calculate gene set scores specific for each sample, and these were further correlated with the expression of the subtype‐specific microRNAs. In ADs, we identified nine aberrantly regulated microRNAs in the terminal respiratory unit (TRU), three in the proximal inflammatory (PI), and nine in the proximal proliferative subtype (PP). In SCCs, 1, 5, 5, and 9 microRNAs were significantly dysregulated in the basal, primitive, classical, and secretory subtypes, respectively. The subtype‐specific microRNAs were highly correlated to specific gene sets, and a distinct pattern of biological processes with high immune activity for the AD PI and SCC secretory subtypes, and upregulation of cell cycle‐related processes in AD PP, SCC primitive, and SCC classical subtypes were found. Several *in silico* predicted targets within the gene sets were identified for the subtype‐specific microRNAs, underpinning the findings. The results were significantly validated in the LUAD (*n* = 492) and LUSC (*n* = 380) TCGA dataset (False discovery rates‐corrected *P*‐value < 0.05). Our study provides novel insight into how expression subtypes determined with discrete biological processes may be regulated by subtype‐specific microRNAs. These results may have importance for the development of combinatory therapeutic strategies for lung cancer patients.

## Graphical abstract

Aberrant expression of microRNAs is an important factor in lung cancer development and differs across different lung cancer expression subtypes. In this study, we identified subtype‐specific microRNAs which may be involved in essential processes characterizing the expression subtypes of ADs and SCCs. Several *in silico* predicted targets were found within the specific processes.

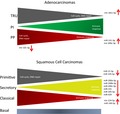

**Abbreviations**
AD: adenocarcinoma
EMT: epithelial–mesenchymal transition
GSE: gene set enrichment
GSVA: gene set variation analysis
PI: proximal inflammatory
PP: proximal proliferative
SCC: squamous cell carcinoma
TRU: terminal respiratory unit

## 1 Introduction

Non‐small‐cell lung cancer (NSCLC) accounts for approximately 85% of all lung cancers, where adenocarcinomas (AD) and squamous cell carcinomas (SCC) are the main histological subtypes (Travis, 2014). AD and SCC origin from different cell types and are associated with different types of mutations. The majority of never‐smoking patients with NSCLC develop AD (Halvorsen *et al.*, 2016; Pikor *et al.*, 2013). The two histological subtypes can be further divided into three and four expression‐based subgroups, respectively. It is shown that the expression subtypes are robust and harbor distinct features. The ADs can be classified as terminal respiratory unit (TRU), proximal inflammatory (PI), and proximal proliferative (PP) using a nearest centroid subtype predictor of 506 genes, as previously described (Hayes *et al.*, 2006; Wilkerson *et al.*, 2012). The TRU subtype, including the majority of never‐smokers, is associated with favorable outcome compared to patients with non‐TRU AD (Ringner *et al.*, 2016). The TRUs are recognized with low mutational burden, but with distinct driver mutations such as *EGFR* mutations, *ALK* rearrangement, and *ROS1* alterations. The PI subtype is described with high immunological activity, high mutational burden, and high frequency of *TP53* mutations. High frequency of *TP53* mutations is also found in the PP subtype in addition to *KRAS* and *STK11* mutations. Increased expression of DNA repair genes is reported, probably reflecting the high number of heavy smokers, found in this subgroup (Network, 2014). The SCC samples can be divided into the four expression subgroups basal, primitive, classical, and secretory based on the previously published centroid classifiers for SCC (Wilkerson *et al.*, 2010). Tumors classified as basal are usually well differentiated and express genes involved in cell adhesion and formation of the basement membrane. The primitive subtype is associated with high proliferation, poor differentiation, and poor prognosis. The classical subtype is also recognized as an aggressive disease and is described as hypermethylated and with high chromosomal instability, probably reflecting the overall high number of heavy smokers. The secretory subtype is characterized with high immune activity and secretory functions (Network, 2012). Based on immune cell estimation, the secretory subtype shares characteristics with normal lung tissue as being immune cell‐rich (Ojlert *et al.*, 2019). Despite demonstrating distinct phenotypic and genetic differences, the molecular mechanisms underlying the development of the expression subtypes are poorly explored.

A class of small noncoding RNA called microRNA has shown to be essential in post‐transcriptional regulation of mRNAs by inhibiting translation or exerting mRNA degradation (Wilczynska and Bushell, 2015). Recently, a pan‐cancer project revealed the role of microRNAs in regulating gene expression signatures of the cancer hallmarks (Dhawan *et al.*, 2018), underpinning the crucial role of microRNAs during tumorigenesis. Aberrant expression of microRNAs is well established as an important factor in lung cancer development, and dysregulation across different histological lung cancer subtypes is reported (Calin and Croce, 2006; Landi *et al.*, 2010; Tran *et al.*, 2018). However, the role of microRNAs in the development of the lung cancer gene expression‐based subtypes is largely unknown.

In this study, we analyzed microRNA expression for a large set of NSCLC samples to identify microRNAs associated with the expression subtypes. Subtype‐specific microRNA expression was correlated to gene set enrichment (GSE) scores in order to identify associated pathways that the microRNAs may be regulating, characterizing the expression subtypes. The results were further validated in independent NSCLC cohorts from The Cancer Genome Atlas (TCGA).

## 2 Materials and methods

### 2.1 Oslo cohort

Patients diagnosed with operable NSCLC from 2006 to 2014 were included in this study (*n* = 241). The patients underwent curatively intended surgical resection at Rikshospitalet, Oslo University Hospital, Norway. Tumor samples were snap‐frozen in liquid nitrogen and stored at −80 °C until RNA isolation was performed. Clinical characteristics are outlined in Table 1. Out of the 241 samples, 132 samples were classified as ADs and 109 as SCC. Never‐smokers were defined as those who had smoked < 100 cigarettes per lifetime. In this study, 19 patients diagnosed with AD were never‐smokers.

**Table 1:** Patient characteristic for the Oslo cohort. Subtype refers to gene expression subtype.

Adenocarcinomas (*n* = 132)		Squamous cell carcinomas (*n* = 109)	
Subtype *n* (%)			
PI	28 (21.2)	Basal	30 (27.5)
PP	23 (17.4)	Primitive	6 (5.5)
TRU	81 (61.4)	Classical	50 (45.9)
		Secretory	23 (21.1)
			
Age (median)	66.6		66.8
Gender female *n* (%)	73 (55.3)		40 (36.7)
Stage *n* (%)			
Ia/Ib	79 (59.8)		56 (51.4)
IIa/IIb	27 (20.5)		36 (33)
IIIa/IIIb	26 (19.7)		17 (15.6)
EGFR mutated *n* (%)	19 (14.4)		na
Pack‐years (mean)	27.6		42.4

The study was approved by the Regional Ethics Committee (S‐05307), and written informed consent was obtained from all patients. The study was performed in agreement with the standards established by the Declaration of Helsinki.

### 2.2 RNA extraction

Total RNA (totRNA) was extracted using standard TRIzol methods (Invitrogen, Carlsbad, CA, USA). RNA quantity and quality (yield, 260/280 ratio and 260/230 ratio) were determined using the NanoDrop ND‐1000 spectrometer (NanoDrop Technologies, Wilmington, DE, USA). The RNA integrity numbers (RIN) were assessed using Agilent 2100 Bioanalyzer and totRNA Nano Kit (Agilent Technologies, Santa Clara, CA, USA) according to the manufacturer's protocol.

### 2.3 mRNA expression analyses

We analyzed mRNA expression from the tumor samples using gene expression microarray from Agilent Technologies (SurePrint G3 Human GE, 8 × 60 K). For the AD samples, we used v.1, whereas for the SCC samples, we used v.3 of the microarray platform. We used 50 ng totRNA as input for the analyses, and the analyses were performed according to the protocol from the supplier. The data are deposited at ArrayExpress with accession number: E‐MTAB‐7954.

### 2.4 microRNA expression analyses

We analyzed microRNA expression using Agilent Human microRNA Microarray for 132 ADs (microarray kit release 16.0, 8 × 60 K) and 109 SCCs (microarray kit release 21.0, 8 × 60 K). We used 100 ng of totRNA in the analyses following the protocol as specified by the manufacturer. The data are deposited at ArrayExpress with accession number: E‐MTAB‐7958.

### 2.5 Normalization of data

Data from the microRNA analyses were log2‐transformed and normalized using the 90th percentile method. The gene expression data were log 2‐transformed and quantile normalized in genespring gx Analysis Software v.12.1 (Agilent Technologies). We filtered out microRNAs detected in < 10% of the AD samples and in < 20% of the SCC samples. After filtering, 562 and 905 microRNAs remained for further analysis, respectively.

### 2.6 Molecular subtyping of adenocarcinomas and squamous cell carcinomas

The AD samples were assigned a gene expression subtype being TRU, PP, or PI, using the previously described 506 gene centroid classifier and Pearson correlation (Wilkerson *et al.*, 2012). The SCC were classified as basal, secretory, primitive, or classical based on the centroid classifier described for SCCs (Wilkerson *et al.*, 2010). Samples negatively correlated with all subtypes were not assigned to any subtype.

### 2.7 Validation dataset

For validation, the lung AD (LUAD) and the lung SCC (LUSC) datasets were obtained from TCGA (Network, 2012, 2014). microRNA and mRNA expression data were extracted as log2(RPKM + 1) values through the Xena browser (https://xenabrowser.net/datapages/). Expression subtyping and gene set variation analysis (GSVA) were performed on mRNA sequencing data from the LUSC (*n* = 553) and the LUAD dataset (*n* = 576). Results from the microRNA analysis were validated in 492 LUAD samples and 380 LUSC samples extracted from TCGA. In addition, we included 45 normal lung tissue samples from the LUAD dataset and 44 normal tissue samples from the LUSC dataset.

### 2.8 Statistics

All statistics were done in r version 3.5.2 (R Development Core Team, 2013). Hierarchical clustering was performed with ComplexHeatmap package version 1.20.0 using ward.D2 as clustering method (Gu *et al.*, 2016). Kruskal–Wallis tests were applied to identify microRNAs differentially expressed between the expression subtypes. Following a significant Kruskal–Wallis test, a *post hoc* Dunn test was utilized to pinpoint in which subtype the microRNA was differentially expressed compared to the others. The packages FSA, fisheries stock analysis R package version 0.8.22 FSA v0.8.22, and Reshape (Wickham, 2007) (Ogle *et al.*, 2018) were utilized. False discovery rates (FDR) were controlled using Benjamini–Hochberg adjustment (Yoav Benjamini, 1995). FDR‐corrected *P*‐values < 0.05 were assigned statistically significant.

Gene set variation analysis is a nonparametric and unsupervised method for assessing GSE in gene expression data. This method allows the evaluation of pathway enrichment for each sample (Hanzelmann *et al.*, 2013). Here, we used the R package GSVA with the Molecular Signatures Database (MSigDB) hallmark gene sets (*n* = 50) downloaded from Broad Institute (http://software.broadinstitute.org/gsea/msigdb/collections.jsp#H) to assess enrichment in the samples. The hallmark gene set contains specific well‐defined biological states or processes and displays coherent expression (Liberzon *et al.*, 2015). An enrichment score for each of the 50 processes was calculated for each sample. Then, Spearman rank correlation was assessed between the enrichment scores and each of the differentially expressed microRNAs. Bonferroni adjustment was applied to the correlation values to correct for multiple testing. Corrected *P*‐values < 0.05 were assigned as statistically significant and further considered. We used miRDIP 4.1 (http://ophid.utoronto.ca/mirDIP/) to identify predicted targets for the subtype‐specific microRNAs (Tokar *et al.*, 2018). This database integrates several computational microRNA–target prediction tools aiming to strengthen the prediction of microRNA/target relationship. Only predictions ranked as very high, corresponding to the top 1% of the list, were accepted as potential targets. Gene sets positively correlated with subtype‐specific microRNAs were not tested. Further, only gene sets with the highest anticorrelated pathway for each microRNA in both cohorts were selected for prediction analyses. Genes were identified as targets for the tested microRNAs if the correlation coefficients were significantly negative (Bonferroni‐corrected *P*‐value < 0.05) in both cohorts.

## 3 Results

**Figure 1:**
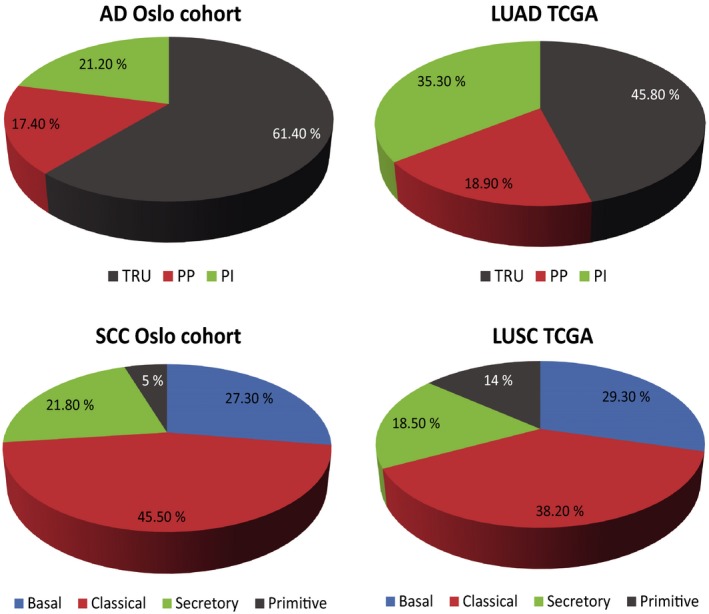
shows the frequency of the expression subtypes for AD and SCC in the Oslo cohort and the TCGA cohort.

The frequencies of the AD subtypes TRU, PI, and PP detected in the Oslo cohort were in line with the TCGA LUAD cohort, although more TRU samples (61.4% versus 45.8%) and fewer PI samples (21.2% versus 35.3%) were identified (Fig. 1). For the SCC samples, a lower frequency of the primitive subtype was detected in the Oslo cohort (5%) than in the TCGA LUSC cohort (14%) as shown in Fig. 1.

**Figure 2:**
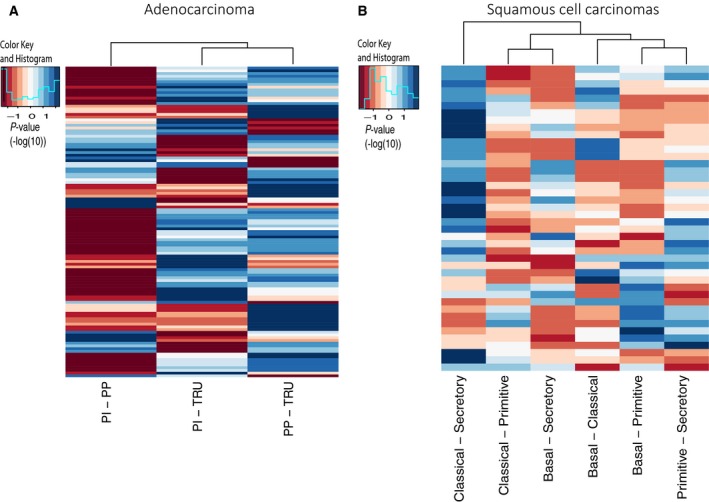
Dunn’s test was applied to explore the microRNA expression between the molecular subtypes. The heatmaps display the *P*‐values (‐log10) from the test, where blue color means significantly different expressed microRNA, and dark‐red means not significantly expressed microRNA. We included the microRNAs that were significantly different in both the Oslo cohort and the TCGA cohorts. A. The *P*‐values obtained from Dunn’s test performed on adenocarcinomas (LUAD). B The *P*‐values obtained from Dunn’s test performed on SCC (LUSC).

We identified 251 microRNAs differentially expressed between the expression subtypes in ADs in the Oslo cohort (FDR‐corrected Kruskal–Wallis *P*‐value < 0.05). Of these, 157 microRNAs were validated in the LUAD cohort of TCGA (FDR‐corrected Kruskal–Wallis *P*‐value < 0.05). Dunn’s test was subsequently used to find expression subtype‐specific microRNAs. In order to be classified as subtype‐specific, the level had to be significantly different from the other subtypes (FDR‐corrected *P*‐value < 0.05) in both cohorts (Oslo and TCGA). As shown in Fig. 2, the number of microRNAs expressed at different levels was highest (low *P*‐values are displayed in blue) when we compared PP and TRU samples. The PI and PP samples showed a more similar microRNA expression pattern.

Next, we included normal lung tissue samples (LUAD, *n* = 45) and focused only on subtype‐specific microRNAs that were also differentially expressed compared to the normal samples. Most of the microRNAs that had similar level as the normal samples were associated with the TRU subtype. These were filtered out. Following these criteria, 21 subtype‐specific microRNAs were identified in ADs (Table 2, Fig. S1, Table S1): three microRNAs characterizing PI (all up), nine microRNAs characterizing PP (two up and seven down), and nine microRNAs characterizing TRU (all up).

**Table 2:** Micrornas significantly differentially expressed between the subtypes and different from normal samples were selected for further analysis. B, basal, C, classical, P, primitive, S, secretory.

Adenocarcinomas			Squamous Cell carcinomas		
Mimat	microRNA	Subtype direction	Mimat	microRNA	Subtype direction
MIMAT0000433	hsa‐miR‐142‐5p	PI high	MIMAT0000089	hsa‐miR‐31‐5p	B high
MIMAT0000434	hsa‐miR‐142‐3p	PI high	MIMAT0000281	hsa‐miR‐224‐5p	C high
MIMAT0000646	hsa‐miR‐155‐5p	PI high	MIMAT0000450	hsa‐miR‐149‐5p	C high
MIMAT0000432	hsa‐miR‐141‐3p	PP high	MIMAT0000764	hsa‐miR‐339‐5p	C high
MIMAT0000617	hsa‐miR‐200c‐3p	PP high	MIMAT0004987	hsa‐miR‐944	C high
MIMAT0000099	hsa‐miR‐101‐3p	PP low	MIMAT0009197	hsa‐miR‐205‐3p	C high
MIMAT0000278	hsa‐miR‐221‐3p	PP low	MIMAT0000226	hsa‐miR‐196a‐5p	P high
MIMAT0001635	hsa‐miR‐452‐5p	PP low	MIMAT0000680	hsa‐miR‐106b‐5p	P high
MIMAT0002809	hsa‐miR‐146b‐5p	PP low	MIMAT0001412	hsa‐miR‐18b‐5p	P high
MIMAT0003266	hsa‐miR‐598‐3p	PP low	MIMAT0000077	hsa‐miR‐22‐3p	P low
MIMAT0004568	hsa‐miR‐221‐5p	PP low	MIMAT0000437	hsa‐miR‐145‐5p	P low
MIMAT0004597	hsa‐miR‐140‐3p	PP low	MIMAT0004550	hsa‐miR‐30c‐2‐3p	S high
MIMAT0000258	hsa‐miR‐181c‐5p	TRU high	MIMAT0000266	miR‐205‐5p	S low
MIMAT0000418	hsa‐miR‐23b‐3p	TRU high	MIMAT0000267	hsa‐miR‐210‐3p	S low
MIMAT0000692	hsa‐miR‐30e‐5p	TRU high	MIMAT0000318	hsa‐miR‐200b‐3p	S low
MIMAT0000758	hsa‐miR‐135b‐5p	TRU high	MIMAT0000432	hsa‐miR‐141‐3p	S low
MIMAT0002871	hsa‐miR‐500a‐3p	TRU high	MIMAT0000617	hsa‐miR‐200c‐3p	S low
MIMAT0003150	hsa‐miR‐455‐5p	TRU high	MIMAT0000682	hsa‐miR‐200a‐3p	S low
MIMAT0003338	hsa‐miR‐660‐5p	TRU high	MIMAT0001080	hsa‐miR‐196b‐5p	S low
MIMAT0004673	hsa‐miR‐29c‐5p	TRU high	MIMAT0001536	hsa‐miR‐429	S low
MIMAT0004775	hsa‐miR‐502‐3p	TRU high			

We applied the same analysis to the SCC samples. Using Kruskal–Wallis test, we identified 50 microRNAs being differentially expressed between the expression subtypes in the Oslo cohort (FDR‐corrected *P*‐value < 0.05). Of these, 41 were validated in the LUSC cohort (FDR‐corrected *P*‐value < 0.05). Dunn’s tests further excluded 21 microRNAs, leaving 20 microRNAs passing the above‐mentioned criteria (Table 2, Fig. S1, Table S1): one microRNA characterizing basal (up), five microRNAs characterizing classical (all up), five microRNAs characterizing primitive (three up and two down), and nine microRNAs characterizing secretory (one up and eight down). Of note, due to the low number of primitive samples in the Oslo cohort, borderline significant microRNAs for tests with this subtype were included if significant in the LUSC cohort. As shown in Fig. 2, the largest difference between the SCC subtypes, in terms of microRNA expression, was found between the secretory and the classical subtypes. On the other side, the classical and the primitive subtypes were most similar with regard to microRNA expression (Fig. 2).

### 3.1 Gene set variation analysis

Gene set variation analysis is a GSE method that estimates underlying pathway activity variation in samples in an unsupervised manner. The hallmark gene sets from MSigDB were used for the analysis (Liberzon *et al.*, 2015). These gene sets contain 50 well‐defined signatures of 50 hallmarks that represent well‐defined biological processes. An enrichment score was calculated sample‐wise for each hallmark without knowledge of any phenotypic information. In order to identify hallmarks associated with subtype‐specific microRNAs, the enrichment scores were correlated with the expression level of the subtype‐specific microRNA using Spearman rank correlation (retaining correlation with a Bonferroni‐corrected *P*‐value < 0.05). As shown in Table 3, immune response, cell cycle maintenance, epithelial–mesenchymal transition (EMT), and metabolism were the processes that correlated the most with the subtype‐specific microRNAs. More details are shown in Table S2.

**Table 3:** Hallmark gene sets significantly associated with high or low expression of subtype‐specific microRNAs in both the Oslo and TCGA cohorts.

Upreg	AD PI	AD PP	AD TRU	
	allograft_rejection	dna_repair	bile_acid_metabolism	
	complement	g2m_checkpoint		
	il2_stat5_signaling	myc_targets_v1		
	il6_jak_stat3_signaling	myc_targets_v2		
	inflammatory_response	glycolysis		
	interferon_alpha_response	oxidative_phosphorylation		
	interferon_gamma_response			
	tnfa_signaling_via_nfkb			

Downreg	AD PI	AD PP	AD TRU	
	NA	allograft_rejection	e2f_targets	
		apoptosis	g2m_checkpoint	
		hedgehog_signaling	spermatogenesis	
		il2_stat5_signaling	myc_targets_v1	
		il6_jak_stat3_signaling	myc_targets_v2	
		inflammatory_response	unfolded_protein_response	
		interferon_alpha_response	mitotic_spindle	
		interferon_gamma_response	mtorc1_signaling	
		tnfa_signaling_via_nfkb	glycolysis	
		p53_pathway		

Upreg	SCC Primitive	SCC Classical	SCC Secretory	SCC Basal
	dna_repair	dna_repair	allograft_rejection	glycolysis
	e2f_targets	p53_pathway	inflammatory_response	estrogen_response_late
	g2m_checkpoint (prog cell cycle)	reactive_oxigen_species_pathway	il6_jak_stat3_signaling	
	mitotic_spindle	e2f_targets	complement	
	spermatogenesis	g2m_checkpoint	epithelial_mesenchymal_transition	
	myc_targets_v1	unfolded_protein_response	angiogenesis	
	myc_targets_v2	spermatogenesis	coagulation	
	oxidative_phosphorylation	myc_targets_v1	myogenesis	
		myc_targets_v2	kras_signaling_up	
		fatty_acid_metabolism		
		glycolysis		
		oxidative_phosphorylation		
		cholesterol_homeostasis		
		peroxisome		
		mtorc1_signaling		
		estrogen_response_late		

Downreg	SCC Primitive	SCC Classical	SCC Secretory	SCC Basal
	allograft_rejection	allograft_rejection	dna_repair	NA
	complement	inflammatory_response	g2m_checkpoint	
	inflammatory_response	complement	e2f_targets	
	il2_stat5_signaling	interferon_alpha_response	unfolded_protein_response	
	il6_jak_stat3_signaling	interferon_gamma_response	spermatogenesis	
	tgf_beta_signaling	il6_jak_stat3_signaling	myc_targets_v1	
	tnfa_signaling_via_nfkb	epithelial_mesenchymal_transition	myc_targets_v2	
	epithelial_mesenchymal_transition	angiogenesis	fatty_acid_metabolism	
	angiogenesis	coagulation	oxidative_phosphorylation	
	coagulation	myogenesis	glycolysis	
	heme_metabolism	kras_signaling_up	cholesterol_homeostasis	
	apoptosis		mtorc1_signaling	
	uv_response_dn			
	myogenesis			
	kras_signaling_up			
	apical_junction			
	androgen_response			

**Figure 3:**
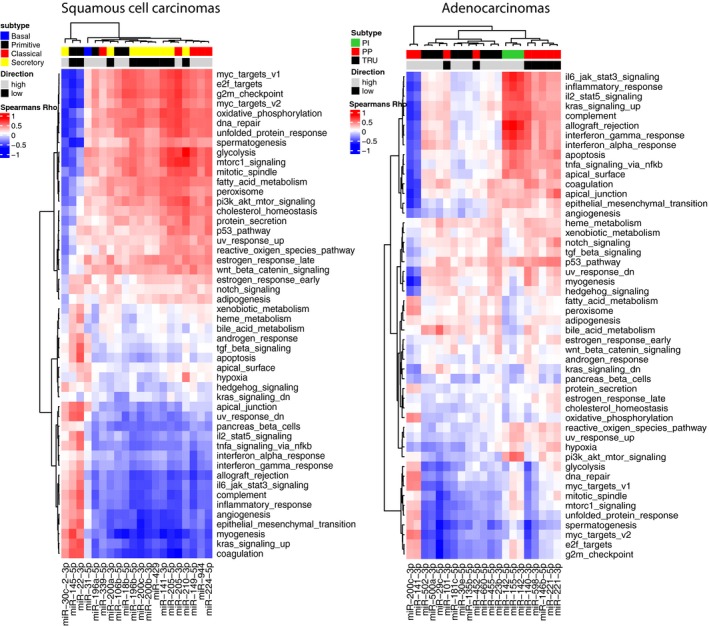
shows the correlation between the subtype‐specific microRNAs and the hallmark gene set for AD and SCC in the Oslo cohort. Subtype annotation indicates which subtype the different microRNAs are associated with. To identify up‐ or downregulated pathways, the correlation coefficient for downregulated microRNAs (annotated with black/low) must be multiplied with −1 (this will switch the red pixels into blue and vice versa).

To further explore and visualize the correlation between subtype‐specific microRNAs and hallmark signatures, the correlation values were hierarchically clustered (Figs 3 and S2).

As displayed in Fig. 3 and Table 3, the AD PI subtype shows upregulation of processes involved in immune response and of the hallmarks cell cycle and DNA repair, which were the opposite of what was found in AD PP subtype. The TRU subtype was associated with upregulation of bile acid metabolism and downregulation of cell cycle and DNA repair. For SCC, a similar pattern was seen with immune response upregulated and cell cycle and DNA repair downregulated in the secretory subtype, just the opposite of what was detected for the primitive and classical subtypes.

In order to assess whether the subtype‐specific microRNA signal originates from the lung cancer cells or from infiltrating immune cells, we compared our subtype‐specific microRNAs with the results from a study investigating human cell‐specific microRNA expression. In this project, the authors sequenced microRNAs from 46 primary cell types, 42 cancer cell lines and tissues (McCall *et al.*, 2017). We extracted the sequencing data from dendritic cells, B lymphocytes, T lymphocytes, macrophages, blood, lung fibroblasts, lung tissue, and lung cancer cell lines. All over, most of the subtype‐specific microRNAs showed highest expression in lung tissue and lung cancer cell lines. Nevertheless, miR‐142‐3p, miR‐142‐5p, and miR 146‐5p were highly expressed in T cells, whereas miR‐140‐3p and miR‐221‐5p were highly expressed in B cells. We also found microRNAs which seemed to be exclusively expressed in lung tissue and lung cancer cell lines. This included 149‐5p, miR‐196a‐5p, miR‐200b‐3p, miR‐224‐5p, miR‐429, and miR‐452‐5p. (Fig. S3, Table S3).

### 3.2 Prediction of targets for the subtype‐specific microRNAs

In order to further elucidate how the subtype‐specific microRNAs may regulate the associated gene sets, we utilized prediction analysis to find potential targets for the microRNAs within the gene sets. First, we identified gene sets being anticorrelated with the subtype‐specific microRNAs. The most anticorrelated gene set for each of the subtype‐specific microRNAs being significant in both cohorts was selected for target analysis. One exception was made; we included *Spermatogenesis* and *E2F targets* for the TRU subtype since these two gene sets were anticorrelated with several of the TRU‐specific microRNAs, but were not ranged as the most anticorrelated gene sets. As expected, for the AD PI, AD TRU, SCC basal, and SCC classical subtypes, no upregulated gene sets were anticorrelated with the selected microRNAs due to only upregulated microRNAs. For AD PP, 11 predicted targets were identified for miR‐101‐3p and miR‐140‐3p within the gene set *G2M checkpoint*, and six predicted targets within inflammatory response were significantly anticorrelated with miR‐200c‐3p and miR‐141‐3p. For SCC secretory subtype, predicted targets for miR‐200a‐3p, miR‐200b‐3p, miR‐200c‐3p, miR‐141‐3p, miR‐ 205‐5p, miR‐429, and miR‐196‐5p were identified within the gene sets inflammatory response (12 predicted targets), *EMT* (32 predicted targets), *myogenesis* (16 predicted targets), *kras signaling up* (13 predicted targets), *il6‐jak‐stat3‐signaling* (four predicted targets), and/or *coagulation* (12 predicted targets). The upregulated gene sets in the SCC primitive subtype *E2F targets* and *G2M checkpoint* were identified with eight and 11 predicted targets for miR‐22‐3p and miR‐145‐5p, respectively. Further, four predicted targets for miR‐106‐5p were found in the downregulated gene sets *myogenesis* and *coagulation*. Three downregulated gene sets were identified with predicted targets for the SCC classical subtype. For more details, see Table S4. Nine of our predicted targets have been functionally validated in previous studies, according to MiRTargetBase (Chou *et al.*, 2018). Details are shown in Table S4.

## 4 Discussion

In this study, we identified 21 and 20 subtype‐specific microRNAs in AD and SCC, respectively. Correlation analysis between microRNA expression and hallmark enrichment scores revealed distinct positive and negative associations with the subtypes of AD and SCC. This suggests that the identified microRNAs may regulate biological processes determining the different subtypes. Even though the identified microRNAs were subtype‐specific, they were involved in many of the same processes, although associated with different targets. We propose that distinct processes characterizing the subtypes, such as high immune activity in AD PI and SCC secretory samples, and proliferation in AD PP, SCC primitive, and SCC classical samples, may be regulated by the identified subtype‐specific microRNAs.

### 4.1 The role of microRNAs in cell cycle and proliferation

Pathways involved in cell cycle and DNA repair signaling were upregulated in SCC classical, SCC primitive, and AD PP subtypes and downregulated in AD TRU and SCC secretory subtypes. These results are in line with previous work where a high proliferation score was detected in the classical, primitive, and PP subtypes (Ojlert *et al.*, 2019). In the SCC classical subtype, all the five classical‐specific microRNAs were upregulated, and subsequently, no anticorrelated predicted targets were detected within the upregulated gene sets. Therefore, we speculate that targets for these microRNAs are inhibitors of the *G2M checkpoint* and *E2F target* gene sets, leading to an increased signaling. For the primitive tumors, the same pathways were upregulated but seem to be controlled in a different manner. With 19 predicted targets of miR‐22‐3p and miR‐145‐5p within *G2M checkpoint* and *E2F target* gene sets, an upregulation of these gene sets may be explained by downregulation of the microRNAs controlling these processes.

In the AD PP subtype, we found 11 predicted targets of miR‐140‐3p and miR‐101‐3p within the *G2M checkpoint* gene set indicating that upregulation of *G2M checkpoint* most likely is a result of repression of miR‐140‐3p and miR‐101‐3p. However, since miR‐101‐3p is expressed both in immune cells and lung tissue, they may be targets of essential components within the cell cycle, simultaneously being involved in the development of suppressive mechanisms in the immune microenvironment. Furthermore, since miR‐140‐3p is highly expressed by B cells, low levels of miR‐140‐3p may indicate the absence of B‐cell infiltration. The TRU tumors were associated with a downregulation of *G2M checkpoint* and *E2F‐targets*. High levels of miR‐181c‐5p may explain this downregulation, supported by identification of 12 predicted targets within these two signaling pathways. None of the 12 predicted targets have been functionally validated, but a recent study showed that miR‐181c‐5p is involved in G2M‐checkpoint regulation, and have direct targets within this pathway (Sun *et al.*, 2019).

We found an upregulation of the *DNA repair* in primitive, classical, and PP subtypes. In addition, the gene set reactive oxygen species were upregulated in the classical subtype. This probably reflects that these subtypes are associated with more heavy smoking (Wilkerson *et al.*, 2010; Wilkerson *et al.*, 2012).

### 4.2 Immune activity regulated by microRNAs

Processes belonging to immunological response were upregulated in AD PI and SCC secretory subtypes and downregulated in AD PP, SCC primitive, and SCC classical subtypes. Interestingly, all three microRNAs associated with the PI subtype are highly expressed in B cells and T cells (Fig. S3), which point to a high immune activity in the PI subtype. This is in line with previous work where high cytolytic and immune score in the PI and secretory subtypes, and low cytolytic and immune score in the PP, primitive, and classical subtypes were detected (Ojlert *et al.*, 2019). Interestingly, in a study of microdissected lung ADs, genes involved in the immune response were not as prominent in the PI subtype as we detected in our study. One possible explanation is that we used bulk tumor, which also harbors cells from the microenvironment such as immune cells (Zabeck *et al.*, 2018). This indicates that the expression subtypes are not restricted to the tumor cell, but can also mirror the tumor microenvironment.

The secretory subtype was recognized with upregulation of immune‐related pathways, which is confirmed in other studies (Faruki *et al.*, 2017; Ojlert *et al.*, 2019). However, in contrast to the PI subtype, the subtype‐specific microRNAs detected in secretory tumors were downregulated (except for miR‐30c‐2‐3p) and mostly associated with expression in lung tissue. Since the secretory subtype is reported to be associated with high immune activity, our findings may indicate that these microRNAs are expressed by the tumor cells, usually suppressing the immune activity. This was further supported when we identified 16 predicted targets within the gene sets *Immune response* and *il6‐jak‐stat3‐signaling*. Interestingly, this was also consistent with the finding of a downregulation of *immune response* in the AD PP subtype, identified with upregulation of two of the same microRNAs (miR‐ 200c‐3p and miR‐141‐3p) which were downregulated in the SCC secretory subtype.

The expression pattern for the secretory subtype shares many similar features to the expression pattern of normal lung tissue. Both normal lung tissue and secretory lung cancer tissue exhibit an immune active pattern. This may explain why many of the secretory subtype‐specific microRNAs were in the same direction as the normal samples.

### 4.3 Epithelial–mesenchymal transition signaling

Several studies have shown that members of the miR‐200 family (miR‐200a, miR‐200b, miR‐200c, miR‐429, and miR‐141) are crucial regulators of the EMT signaling (Humphries and Yang, 2015). It has been shown that miR‐200 family can target ZEB1 and ZEB2 and promote expression of E‐cadherin, thus hinder migration, invasion, and tumor angiogenesis (Korpal *et al.*, 2008).Tumors with the secretory subtype were recognized with a downregulation of all members of the miR‐200 family and upregulation of *EMT* and *angiogenesis*. For *EMT signaling*, 32 predicted targets for the miR‐200 family were identified within our dataset supporting these findings. Interestingly, in a study of pan‐cancer EMT signature, immune cell signaling was strongly correlated to EMT (Mak *et al.*, 2016). This was also shown with the clustering of microRNAs and the hallmark gene sets (Fig. 3) where EMT‐ and immune‐related processes were identified in the same subcluster. This may explain why the classical and primitive subtypes revealed a downregulation of *EMT* and *angiogenesis*, in addition to a low immune activity. An opposite result was discovered for the SCC secretory subtype, identified with upregulation of the same pathways. Further, an association between *EMT* and increased expression of PD‐L1 has been reported, and *EMT* has been suggested in regulating immune escape in lung cancer (Chen *et al.*, 2014). However, we did not observe high PD‐L1 expression in the secretory subtype in our previous study (Ojlert *et al.*, 2019).

There are some limitations in this study. Due to fewer samples included in the Oslo cohort, microRNAs with borderline significance were included if significant in the TCGA cohort. This may implicate that other microRNAs important for this subtype were not captured during the first analysis. However, all the results were validated in TCGA which make the findings in this study robust and repeatable. Furthermore, only one microRNA (miR‐31‐5p) was significantly associated with the basal subtype, resulting in few pathways correlated with this group. This may indicate that development of basal tumors is driven by other mechanisms than microRNAs, or that some basal‐specific microRNAs were not captured by our analyses. However, we found that miR‐31‐5p was highly lung cancer and lung tissue specific which may indicate that this microRNA is oncogenic and plays a specific role in basal lung tumors.

The samples from the Oslo cohort were analyzed using a microarray platform, whereas the TCGA samples were profiled using next‐generation sequencing technology. Thus, there may exist additional subtype‐specific microRNAs which were not captured in this study as the microRNAs present on the microarray defined the microRNA focus. Nevertheless, the resulting subtype‐specific microRNAs reported in the present study were robustly identified across platforms.

## 5 Conclusions

In this study, we showed that subtype‐specific microRNAs may be involved in essential processes characterizing the expression subtypes of ADs and SCCs. Of note, functional studies are warranted in order to detect precise targets for the subtype‐specific microRNAs. Unraveling the underlying biology in lung cancer subtypes may be important in order to offer the patients a more stratified targeted therapy. Inhibition of essential pathways together with standard care of treatment including immunotherapy may be a beneficial strategy.

## Conflict of interest

The authors declare no conflict of interest.

## Author contributions

MRA, ÅH, and ARH conceived and design the project. ARH acquired the data. ARH, MRA, DN, and ÅKÕ analyzed and interpreted the data. ARH, ÅH, and MRA wrote the paper. SS, ÅH, and OTB collected the clinical data and the material, and participated in the evaluation of the data. All authors read, revised the manuscript critically, and approved the final manuscript.

## Supporting information


**Fig. S1** **. **Examples of microRNAs significantly associated with one specific expression subtype shown for the LUAD and LUSC cohorts.

**Fig. S2** **. **This figure shows the correlation between the subtype‐specific microRNAs and the hallmark gene set for AD and SCC in the TCGA cohort. Subtype annotation indicate which subtype the different microRNAs are associated with. To identify up‐ or down‐regulated pathways, the correlation‐coefficient for downregulated microRNAs (annotated with black/low) must be multiplied with ‐1 (this will switch the red pixels into blue and vice versa).

**Fig. S3** **. **This figure shows the expression of the identified subtype‐specific microRNAs across different immune cells, lung tissue and lung cancer cell lines. Data is extracted from McCall et al.^24^ The color‐bars show the subtype and direction associated with the microRNA. Three microRNAs (miR‐141‐3p, miR‐145‐5p, miR‐200c‐3p) were specific to subtypes both in AD and SCC, and are annotated with the suffix 2.

**Table S1** **.** Dunn test was utilized to identify expression subtype‐specific microRNAs in Oslo Cohort and TCGA.

**Table S2** **. **Shows the hallmark gene sets associated with subtype‐specific microRNAs.

**Table S3** **. **Shows which cells the subtype‐specific microRNA may originate from. The sequencing data is from a project were the authors sequenced microRNAs from 46 primary cell types, 42 cancer cell lines and tissues (McCall *et al.*, 2017). We extracted the sequencing data for the subtype‐specific microRNAs.

**Table S4** **. **Shows in silico predicted targets for subtype‐specific microRNAs within the associated gene set. Genes marked with a star are previous functionally validated according to MiRTargetBase (Chou *et al*., 2018).

## References

[mol212571-bib-0001] Calin GA and Croce CM (2006) MicroRNA signatures in human cancers. Nat Rev Cancer 6, 857–866. 17060945 10.1038/nrc1997

[mol212571-bib-0002] Cancer Genome Atlas Research Network (2012) Comprehensive genomic characterization of squamous cell lung cancers. Nature 489, 519–525. 22960745 10.1038/nature11404 PMC3466113

[mol212571-bib-0003] Cancer Genome Atlas Research Network (2014) Comprehensive molecular profiling of lung adenocarcinoma. Nature 511, 543–550. 25079552 10.1038/nature13385 PMC4231481

[mol212571-bib-0004] Chen L , Gibbons DL , Goswami S , Cortez MA , Ahn YH , Byers LA , Zhang X , Yi X , Dwyer D , Lin W *et al* (2014) Metastasis is regulated via microRNA‐200/ZEB1 axis control of tumour cell PD‐L1 expression and intratumoral immunosuppression. Nat Commun 5, 5241. 25348003 10.1038/ncomms6241 PMC4212319

[mol212571-bib-0005] Chou CH , Shrestha S , Yang CD , Chang NW , Lin YL , Liao KW , Huang WC , Sun TH , Tu SJ , Lee WH *et al* (2018) miRTarBase update 2018: a resource for experimentally validated microRNA‐target interactions. Nucleic Acids Res 46, D296–d302. 29126174 10.1093/nar/gkx1067 PMC5753222

[mol212571-bib-0006] Dhawan A , Scott JG , Harris AL and Buffa FM (2018) Pan‐cancer characterisation of microRNA across cancer hallmarks reveals microRNA‐mediated downregulation of tumour suppressors. Nat Commun 9, 5228. 30531873 10.1038/s41467-018-07657-1 PMC6286392

[mol212571-bib-0007] Faruki H , Mayhew GM , Serody JS , Hayes DN , Perou CM and Lai‐Goldman M (2017) Lung adenocarcinoma and squamous cell carcinoma gene expression subtypes demonstrate significant differences in tumor immune landscape. J Thoracic Oncol 12, 943–953. 10.1016/j.jtho.2017.03.010 PMC6557266 28341226

[mol212571-bib-0008] Gu Z , Eils R and Schlesner M (2016) Complex heatmaps reveal patterns and correlations in multidimensional genomic data. Bioinformatics 32, 2847–2849. 27207943 10.1093/bioinformatics/btw313

[mol212571-bib-0009] Halvorsen AR , Silwal‐Pandit L , Meza‐Zepeda LA , Vodak D , Vu P , Sagerup C , Hovig E , Myklebost O , Børresen‐Dale A‐L , Brustugun OT *et al* (2016) TP53 mutation spectrum in smokers and never smoking lung cancer patients. Front Genet 7, 85. 27242894 10.3389/fgene.2016.00085 PMC4863128

[mol212571-bib-0010] Hanzelmann S , Castelo R and Guinney J (2013) GSVA: gene set variation analysis for microarray and RNA‐seq data. BMC Bioinformat 14, 7. 10.1186/1471-2105-14-7 PMC3618321 23323831

[mol212571-bib-0011] Hayes DN , Monti S , Parmigiani G , Gilks CB , Naoki K , Bhattacharjee A , Socinski MA , Perou C and Meyerson M (2006) Gene expression profiling reveals reproducible human lung adenocarcinoma subtypes in multiple independent patient cohorts. J Clin Oncol 24, 5079–5090. 17075127 10.1200/JCO.2005.05.1748

[mol212571-bib-0012] Humphries B and Yang C (2015) The microRNA‐200 family: small molecules with novel roles in cancer development, progression and therapy. Oncotarget 6, 6472–6498. 25762624 10.18632/oncotarget.3052 PMC4466628

[mol212571-bib-0013] Korpal M , Lee ES , Hu G and Kang Y (2008) The miR‐200 family inhibits epithelial‐mesenchymal transition and cancer cell migration by direct targeting of E‐cadherin transcriptional repressors ZEB1 and ZEB2. J Biol Chem 283, 14910–14914. 18411277 10.1074/jbc.C800074200 PMC3258899

[mol212571-bib-0014] Landi MT , Zhao Y , Rotunno M , Koshiol J , Liu H , Bergen AW , Rubagotti M , Goldstein AM , Linnoila I , Marincola FM *et al* (2010) MicroRNA expression differentiates histology and predicts survival of lung cancer. Clin Cancer Res 16, 430–441. 20068076 10.1158/1078-0432.CCR-09-1736 PMC3163170

[mol212571-bib-0015] Liberzon A , Birger C , Thorvaldsdottir H , Ghandi M , Mesirov JP and Tamayo P (2015) The Molecular Signatures Database (MSigDB) hallmark gene set collection. Cell Syst 1, 417–425. 26771021 10.1016/j.cels.2015.12.004 PMC4707969

[mol212571-bib-0016] Mak MP , Tong P , Diao L , Cardnell RJ , Gibbons DL , William WN , Skoulidis F , Parra ER , Rodriguez‐Canales J *et al* (2016) A patient‐derived, pan‐cancer EMT signature identifies global molecular alterations and immune target enrichment following epithelial‐to‐mesenchymal transition. Clin Cancer Res 22, 609–620. 26420858 10.1158/1078-0432.CCR-15-0876 PMC4737991

[mol212571-bib-0017] McCall MN , Kim MS , Adil M , Patil AH , Lu Y , Mitchell CJ , Leal‐Rojas P , Xu J , Kumar M , Dawson VL *et al* (2017) Toward the human cellular microRNAome. Genome Res 27, 1769–1781. 28877962 10.1101/gr.222067.117 PMC5630040

[mol212571-bib-0018] Ogle DH , Wheeler P and Dinno A (2018) FSA: Fisheries stock analysis.

[mol212571-bib-0019] Ojlert AK , Halvorsen AR , Nebdal D , Lund‐Iversen M , Solberg S , Brustugun OT , Lingjærde OC and Helland Å (2019) The immune microenvironment in non‐small cell lung cancer is predictive of prognosis after surgery. Mol Oncol 13, 1166–1179. 30854794 10.1002/1878-0261.12475 PMC6487716

[mol212571-bib-0020] Pikor LA , Ramnarine VR , Lam S and Lam WL (2013) Genetic alterations defining NSCLC subtypes and their therapeutic implications. Lung Cancer 82, 179–189. 24011633 10.1016/j.lungcan.2013.07.025

[mol212571-bib-0021] R Development Core Team (2013) R: Language and Environment for Statistical Computing. R Foundation for Statistical Computing, Vienna, Austria http://www.R-project.org/

[mol212571-bib-0022] Ringner M , Jonsson G and Staaf J (2016) Prognostic and chemotherapy predictive value of gene‐expression phenotypes in primary lung adenocarcinoma. Clin Cancer Res 22, 218–229. 26265693 10.1158/1078-0432.CCR-15-0529

[mol212571-bib-0023] Sun Z , Li Y , Wang H , Cai M , Gao S , Liu J , Tong L , Hu Z K *et al* (2019) miR‐181c‐5p mediates simulated microgravity‐induced impaired osteoblast proliferation by promoting cell cycle arrested in the G2 phase. J Cell Mol Med 23, 3302–3316. 30761733 10.1111/jcmm.14220 PMC6484313

[mol212571-bib-0024] Tokar T , Pastrello C , Rossos AEM , Abovsky M , Hauschild AC , Tsay M , Lu R and Jurisica I (2018) mirDIP 4.1‐integrative database of human microRNA target predictions. Nucleic Acids Res 46, D360–d370. 29194489 10.1093/nar/gkx1144 PMC5753284

[mol212571-bib-0025] Tran N , Abhyankar V , Nguyen K , Weidanz J and Gao J (2018) MicroRNA dysregulational synergistic network: discovering microRNA dysregulatory modules across subtypes in non‐small cell lung cancers. BMC Bioinformat 19, 504. 10.1186/s12859-018-2536-0 PMC6302368 30577741

[mol212571-bib-0026] Travis WD (2014) The 2015 WHO classification of lung tumors. Der Pathologe 35(Suppl 2), 188. 25394966 10.1007/s00292-014-1974-3

[mol212571-bib-0027] Wickham H (2007) Reshaping Data with the reshape Package. J Statistic Softw Artic 21, 1–20.

[mol212571-bib-0028] Wilczynska A and Bushell M (2015) The complexity of miRNA‐mediated repression. Cell Death Differ 22, 22–33. 25190144 10.1038/cdd.2014.112 PMC4262769

[mol212571-bib-0029] Wilkerson MD , Yin X , Hoadley KA , Liu Y , Hayward MC , Cabanski CR , Muldrew K , Miller CR , Randell SH , Socinski MA *et al* (2010) Lung squamous cell carcinoma mRNA expression subtypes are reproducible, clinically important, and correspond to normal cell types. Clin Cancer Res 16, 4864–4875. 20643781 10.1158/1078-0432.CCR-10-0199 PMC2953768

[mol212571-bib-0030] Wilkerson MD , Yin X , Walter V , Zhao N , Cabanski CR , Hayward MC , Miller CR , Socinski MA , Parsons AM , Thorne LB *et al* (2012) Differential pathogenesis of lung adenocarcinoma subtypes involving sequence mutations, copy number, chromosomal instability, and methylation. PLoS ONE 7, e36530. 22590557 10.1371/journal.pone.0036530 PMC3349715

[mol212571-bib-0031] Yoav Benjamini YH (1995). Controlling the false discovery rate: a practical and powerful approach to multiple testing. J Royal Statist Soc B 57, pp. 289–300

[mol212571-bib-0032] Zabeck H , Dienemann H , Hoffmann H , Pfannschmidt J , Warth A , Schnabel PA , Muley T , Meister M , Sültmann H , Fröhlich H *et al* (2018) Molecular signatures in IASLC/ATS/ERS classified growth patterns of lung adenocarcinoma. PLoS ONE 13, e0206132. 30352093 10.1371/journal.pone.0206132 PMC6198952

