# Supplementary figures and images for: Identification of microRNAs involved in pathways which characterize the expression subtypes of NSCLC
Source: Mol Oncol. 2019 Sep 22;13(12):2604–15. doi: 10.1002/1878-0261.12571 (PMC6887593; doi:10.1002/1878-0261.12571)

# Squamous cell carcinomas

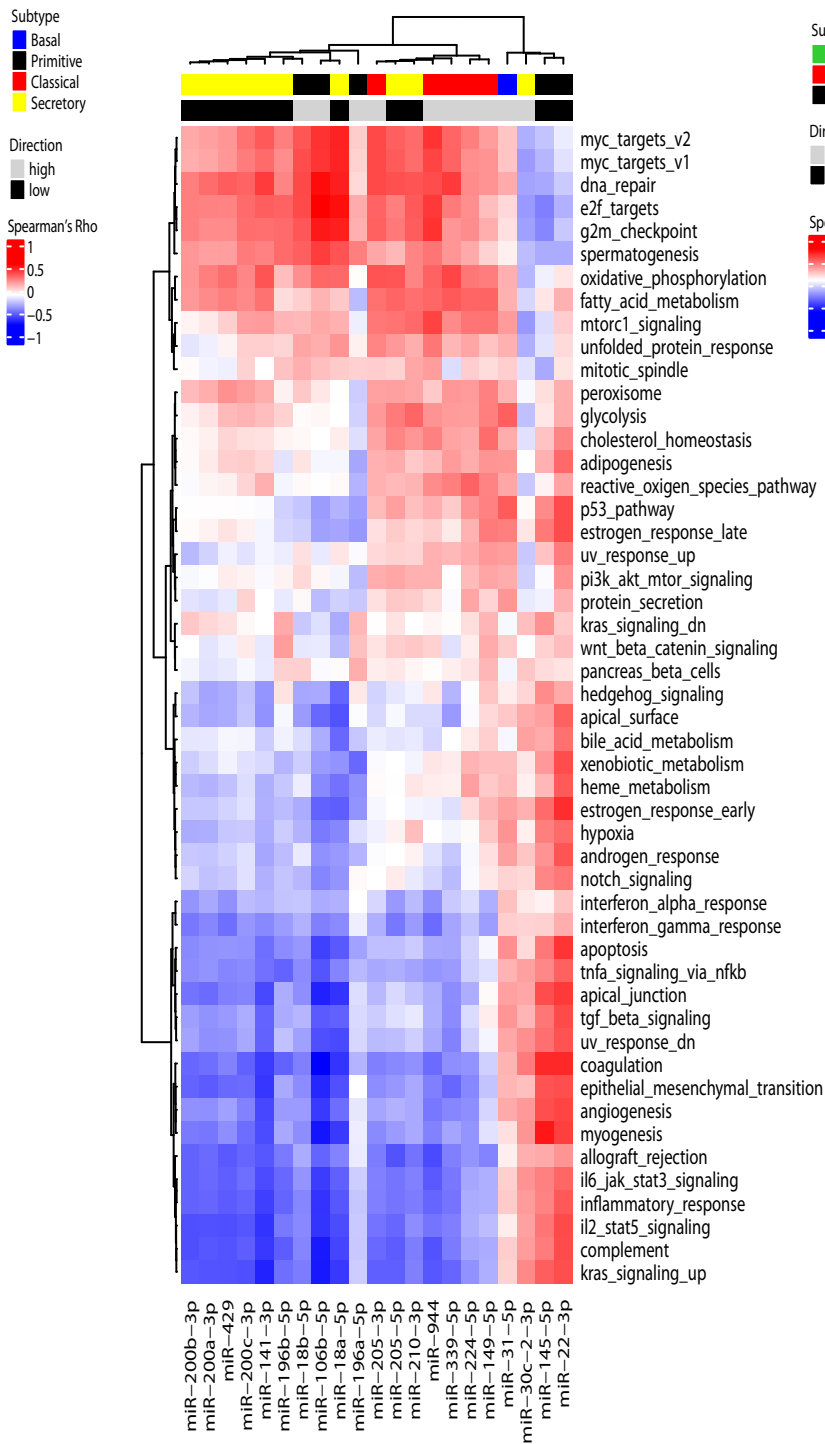

# Adenocarcinomas

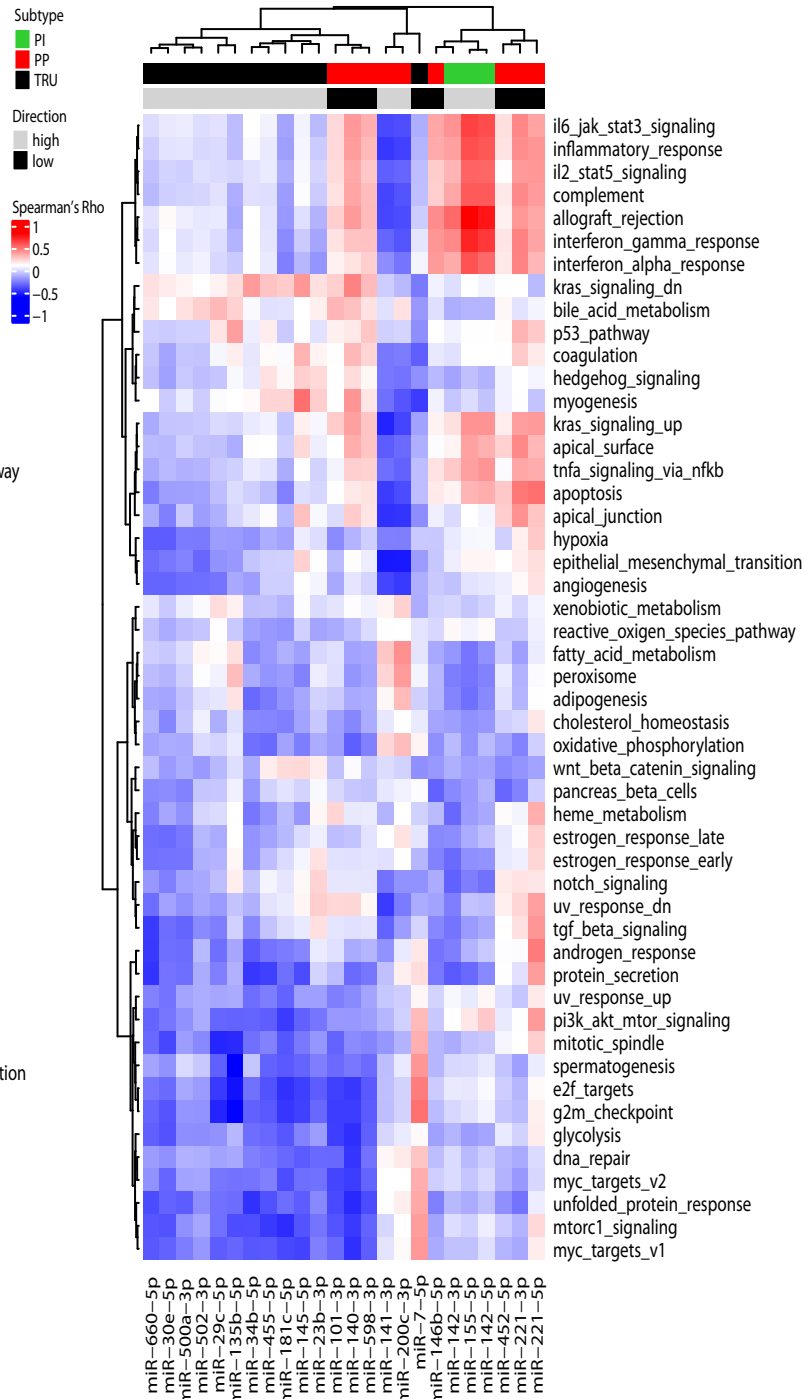

Supplement: Supplementary file 2 — Fig. S2 . This figure shows the correlation between the subtype‐specific microRNAs and the hallmark gene set for AD and SCC in the TCGA cohort. Subtype annotation indicate which subtype the different microRNAs are associated with. To identify up‐ or down‐regulated pathways, the correlation‐coefficient for downregulated microRNAs (annotated with black/low) must be multiplied with ‐1 (this will switch the red pixels into blue and vice versa). [file MOL2-13-2604-s002.pdf]

Adenocarcinomas

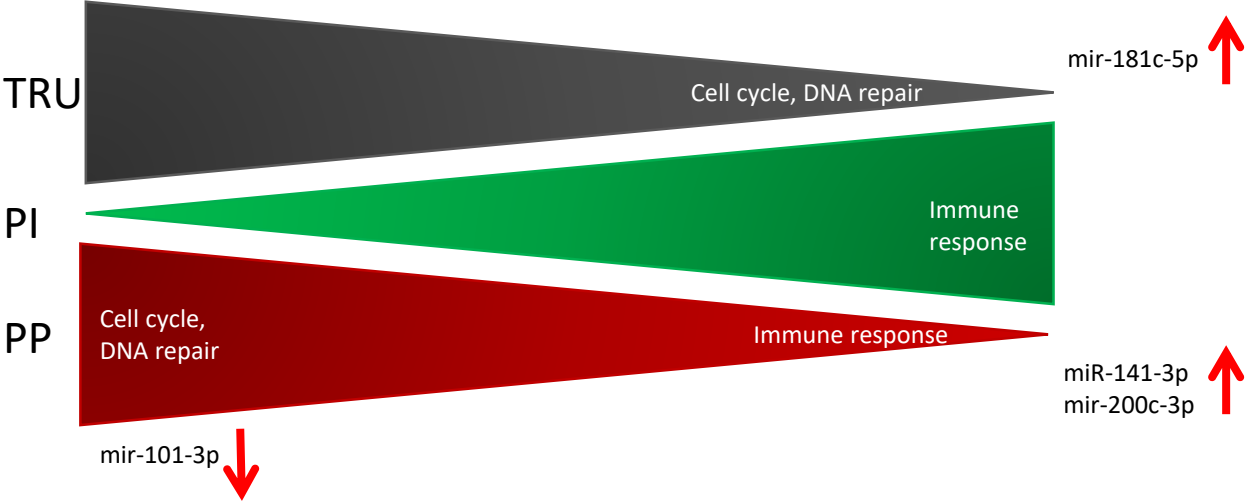

Squamous Cell Carcinomas

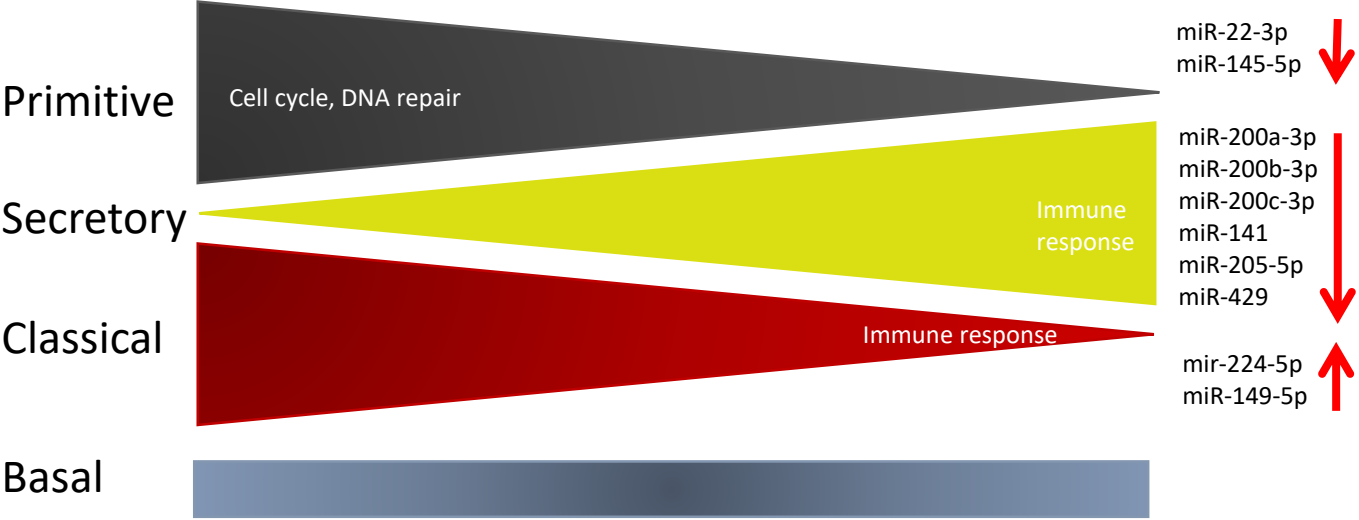

Supplement: Supplementary file 8 [file MOL2-13-2604-s007.pdf]
